# A stronger association of depression with rheumatoid arthritis in presence of obesity and hypertriglyceridemia

**DOI:** 10.3389/fepid.2023.1216497

**Published:** 2023-12-15

**Authors:** Grayden Shand, Daniel T. Fuller, Leon Lufkin, Carly Lovelett, Nabendu Pal, Sumona Mondal, Shantanu Sur

**Affiliations:** ^1^ David D. Reh School of Business, Clarkson University, Potsdam, NY, United States; ^2^ Department of Mathematics, Clarkson University, Potsdam, NY, United States; ^3^ The Clarkson School, Clarkson University, Potsdam, NY, United States; ^4^ Department of Statistics and Data Science, Yale University, New Haven, CT, United States; ^5^ Saint Lawrence Health, Canton Potsdam Hospital, Potsdam, NY, United States; ^6^ Department of Mathematics, University of Louisiana at Lafayette, Lafayette, LA, United States; ^7^ Department of Biology, Clarkson University, Potsdam, NY, United States

**Keywords:** comorbidities, synergistic effect, inflammation, c-reactive protein, interactions, depression, NHANES

## Abstract

**Background:**

Rheumatoid arthritis (RA) is an autoimmune disorder characterized by chronic and systemic inflammation. Recent research underscores the role of chronic inflammation in multiple common RA comorbidities such as depression, obesity, and cardiovascular diseases (CVDs), suggesting a potential overlap of the pathogenic mechanisms for RA. However, it is not well understood how the coexistence of these comorbid conditions impacts the risk of RA and whether any such association relates to body's inflammatory state.

**Methods:**

We used data from the 2007-2010 United States National Health and Nutrition Examination Survey (NHANES) database and compared RA prevalence between subsamples with the presence of any two conditions among depression, obesity, and hypertriglyceridemia (HTG). Each subsample was further divided into three categories based on the serum level of the inflammatory marker C-reactive protein (CRP) and analyzed for statistically significant differences using three-way *χ*^2^ tests of independence.

**Results:**

The study was conducted on 4,136 patients who fulfilled the inclusion criteria (representing 163,540,241 individuals after adjustment for sampling weights). Rates of depression, obesity, and HTG were found to be significantly higher (*P* < 0.001) among the subjects with RA compared with the control population with no arthritis. The presence of depression along with obesity or HTG showed a noticeably higher RA prevalence but such an association was not observed for the combination of obesity and HTG. The synergistic effect of HTG with depression was found to be most prominent at a medium CRP level (1–3 mg/L), while for obesity, the effect was observed across all CRP levels examined. These findings were further confirmed by the three-way *χ*^2^ test for independence.

**Conclusions:**

The presence of obesity or HTG in subjects suffering from depression might pose an increased risk of RA. Inflammatory mechanisms potentially play an important underlying role as suggested by the strong dependency of the association to CRP level. Identification of synergistic associations between RA risk conditions could provide useful information to predict the development and progress of RA.

## 1. Introduction

Rheumatoid Arthritis (RA) is a chronic progressive autoimmune disease affecting an estimated 0.5%–1% of adults in North America (1, 2). It primarily affects the synovial joints and is characterized by stiffness, major joint pain, and irreversible joint deformity causing serious disability if left untreated. The prevalence of RA varies across age, gender, and ethnicity with a number of genetic, environmental, and behavioral factors known to increase disease risk (1, 3–6). RA often associates with a variety of comorbid conditions including depression, obesity, cardiovascular diseases (CVDs), and diabetes (7–10), suggesting an overlap of RA pathogenic mechanisms with these chronic conditions. The existence of a shared or overlapping pathway of pathogenesis would not only increase the prevalence of comorbidities among RA patients but could potentially increase the risk of RA when one or more comorbidities are present in a patient. Since chronic, systemic inflammation with dysregulated immunity is a hallmark of RA and underlies most of its clinical manifestations, we speculate that comorbidities with a known inflammatory link would be highly pertinent in this context (11). While many different comorbidities among RA subjects have been studied in detail, less information is available on whether the presence of multiple comorbidities poses a risk for the development or progress of RA.

Depression is identified as a major RA comorbidity with a prevalence of 13%–42% in RA patients and is associated with a poorer outcome with increased pain, disability, and risk of mortality (12–14). Analyzing longitudinal data from the National Health Insurance Research Database of Taiwan, Lu et al. reported that the incidence of depression among RA patients was 15.69 per 1,000 person-years (PYs) compared with 8.95 per 1,000 PYs in the control population (15). Interestingly, this study also found the incidence of RA to be significantly higher in the population with depression than in the control population, suggesting a bidirectional relationship between RA and depression (15). Similar to depression, obesity is reported to have a higher prevalence among RA patients, and thus, given the adverse impact of obesity on multiple chronic diseases, one major focus of research is to understand how obesity influences the course of RA (16, 17). Studies have linked obesity to worsened disease outcomes in RA and also found that obese patients had lower odds of achieving and sustaining remission when compared with non-obese patients (18, 19). Although the role of obesity in RA development has not been established fully, the results from several longitudinal studies indicate obesity increases the risk of RA (17). Other conditions such as hypertriglyceridemia (HTG), a condition when the serum triglyceride concentration exceeds a certain level (150 mg/dl), are also important from the context of their interactions with other RA-associated conditions (20). Specifically, HTG is associated with increased atherosclerosis formation (21), and is a well-known risk factor for many CVDs (22). Risk of several CVDs including coronary artery disease and peripheral vascular disease as well as major CVD events such as myocardial infarction and stroke are substantially increased in RA, resulting in higher CVD-related death (23–25). While HTG has not been shown to be an important risk factor for RA, it is associated with increased severity of symptoms, potentially related to its relationship to CVDs.

One major hallmark of RA is chronic inflammation associated with a dysregulation of the innate and adaptive immune systems (26). Stimulation of inflammatory pathways has also been reported for depression, obesity, and HTG. For example, excess fat tissue in obese individuals is associated with high levels of inflammatory cytokines, including tumor necrosis factor alpha (TNF-α), interleukin 1 (IL-1) 1, interleukin 6 (IL-6), and C-reactive protein (CRP), all of which are known mediators of RA (27, 28). Similarly, higher levels of TNF-α, IL-1, IL-6, and CRP are reported in subjects with increased depression (29). HTG is also associated with increased inflammation (manifested by increased levels of CRP, IL-6, and other inflammatory markers) (30), which is partially attributed to a reduced anti-inflammatory capacity of serum high-density lipoproteins resulting from a higher-than-average triglyceride content (31). The higher level of inflammatory markers in these conditions and RA suggest inflammation plays an important role in the development of these diseases. Moreover, the frequent occurrence of these conditions in subjects with RA is suggestive of a common molecular underpinning potentially mediated through inflammatory mechanisms (26).

The objective of this work is to investigate any potential relationship between depression, obesity, and HTG—each of which independently relates to chronic inflammation—in their association with RA. Using publicly available data from National Health and Nutrition Examination Survey (NHANES), we explored whether the coexistence of any two of these conditions synergistically enhances the association with RA. Additionally, considering their common connection with inflammation, we also studied if such an association is related to the general inflammatory state of the subject.

## 2. Materials and methods

### 2.1. Study population

The CDC-operated online portal NHANES offers detailed health data, obtained through interviews and physical examinations, on samples drawn from the US population. It employs a stratified, multistage, nationally representative cross-sectional survey design in order to provide countrywide inference. The survey strategy oversamples from select smaller subpopulations such as Hispanic people, low-income people, and adults aged 60 and over, in order to obtain more precise information on health status in these groups, and subsequently uses weights to correct for any bias due to selective oversampling (32). The survey protocol and data collection methods of NHANES data used in this study were approved by the National Center for Health Statistics Ethics Review Board (protocol #2005-06). The types of data collected by NHANES include demographic variables, socioeconomic condition (SEC), questionnaires, and bio-specimen examination. The participants are represented by a unique number in each dataset to protect their identity.

### 2.2. Data collection

Analyses were conducted on data from the 2007-2008 and the 2009-2010 NHANES datasets to examine the relationship of inflammation (measured by CRP) with the three primary factors of interest: depression, obesity, and triglyceride. These years were chosen because of their detailed information on CRP, which is not present in later years of NHANES data. Response rates for participation in both interviews and physical examinations were close for each two-year cycle and ranged from 75% to 80%. Subjects younger than 18 years were excluded from the analysis to prevent inclusion of juvenile RA and subjects with ages greater than 79 years were excluded as NHANES does not differentiate ages 80 years or above.

### 2.3. Variables and measurements

#### 2.3.1. RA and comorbid conditions

##### 2.3.1.1. RA

RA was defined based on answers to the following questions: (1) “Has a doctor or other health professional ever told that you had arthritis?” If the response to this question is yes, the second question was (2) “What type of arthritis?” Subjects with the response of “Rheumatoid Arthritis” were considered to have RA.

##### 2.3.1.2. Depression

A nine-item depression screening instrument built on Patient Health Questionnaire (PHQ-9) was used to assess depression. PHQ-9 scores can range from 0 to 27 and we considered subjects with a score of ≥10 to suffer from depression, which includes the categories of moderate-severe, and severe depression (33).

##### 2.3.1.3. Hypertension

Three consecutive individual blood pressure measurements 30 s apart were obtained by certified examiners using a sphygmomanometer after the participants had been seated and had rested for at least five minutes. A fourth measurement was taken if any of the previous three were missing or performed erroneously. The means of these recorded values were used to represent the participants’ systolic and diastolic blood pressures. Systolic and diastolic hypertension were defined when the measured systolic and diastolic blood pressure were ≥130 mm Hg and ≥80 mm Hg, respectively (34).

##### 2.3.1.4. Hypertriglyceridemia (HTG)

Triglyceride measurements were obtained with the Roche/Hitachi Modular *P* Chemistry Analyzer method. Serum triglyceride concentration ≥150 mg/dl was considered as HTG in accordance with standards set by the Adult Treatment Panel III of the National Cholesterol Education Program (20).

##### 2.3.1.5. Obesity

Height was measured using a wall-mounted stadiometer, and weight was measured using a Toledo digital scale. BMI was calculated from standing height and body weight measurements. Participants with BMI ≥30 kg/m^2^ were considered obese (35).

##### 2.3.1.6. Diabetes

Participants were recorded as diabetic if they self-reported the condition or had hemoglobin A1c ≥6.5%. Self-reported diabetes was determined from the participant answering “yes” to at least one of a set of questions regarding physician diagnosis and insulin regulation.

Stroke: Stroke was defined as a self-reported history of stroke.

#### 2.3.2. Other variables

##### 2.3.2.1. CRP

CRP measurements in serum samples were obtained using latex-enhanced nephelometry. Measured CRP levels were further divided into three risk categories, namely low (<1 mg/L), medium (1–3 mg/L), and high (> than 3 mg/L) based on the guideline provided by the Centers for Disease Control and Prevention (CDC) and the American Heart Association (AHA) (36).

Demographics: Age, sex, and median family incomes were obtained from the NHANES database.

### 2.4. Statistical analysis

The NHANES data were adjusted by implementing appropriate sampling weights according to the National Center for Health Statistics analytical guidelines to account for the potential bias created by the sampling procedure (32). We presented here the analyses both with and without adjusting the data according to the sample weights.

All *P*-values presented here are from two-sided tests. *P*-values less than 0.05 were considered statistically significant. The inter-group difference in abundance was evaluated using a two-way chi-square (*χ*^2^) test conducted on unweighted data. Three-way *χ*^2^ tests were used to test for significant differences in group proportions of subpopulations and were performed on weighted data scaled down to the sample size of the unweighted data. Residual analysis was utilized to identify the degree of contribution to the test statistic from each categorical combination.

Data preprocessing and statistical analyses were performed using R software version 3.2.5 and all plots were generated using the ggplot2 package.

## 3. Results

**Figure 1:**
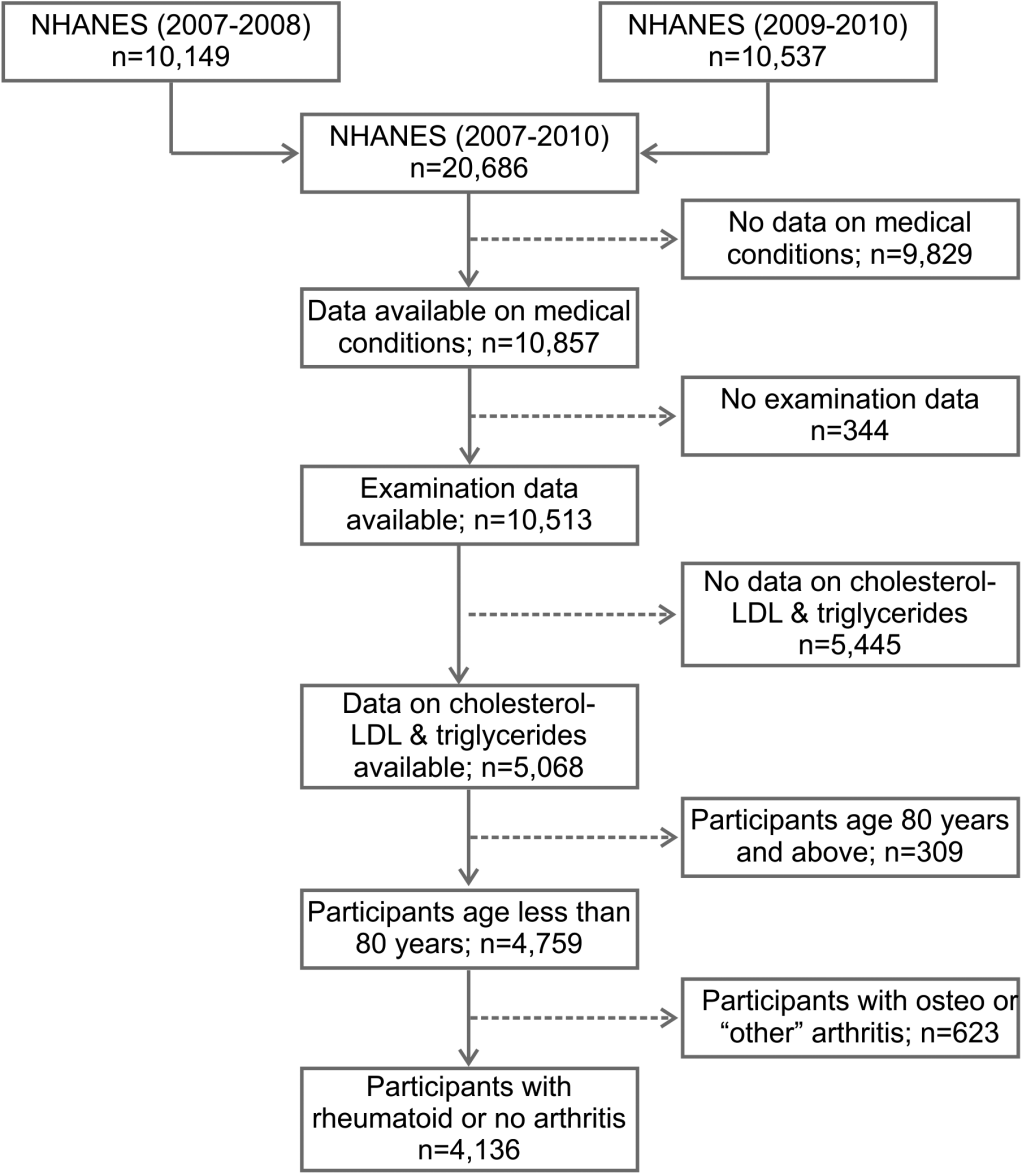
Flow diagram of the selection of study population from NHANES data (2007–2010). NHANES, National Health and Nutrition Examination Survey; LDL, low-density lipoproteins.

The NHANES study included 10,149 participants in 2007–2008 and 10,537 participants in 2009–2010. The samples from these two survey cycles were considered as non-overlapping, and therefore, independent observations. Of the 20,686 participants from 2007 to 2010, a total of 4,136 participants satisfied the inclusion criteria (Figure 1), of which 305 were positive for RA. Table 1 shows a comparison of demographics and major comorbidities between the RA and non-RA study populations (unweighted *n* = 4,136; weighted *N* = 163,540,241). The comparisons were made for both unweighted and weighted data to check for any potential bias arising from the oversampling of select subpopulations. We observed the values obtained from unweighted and weighted data to corroborate well, ruling out the potential for any substantial sampling bias. Age and gender distributions were found to be significantly different between these two groups. 50.2% of the RA population belonged to the age group of more than 60 years and 40% belonged to the age group of 41–60 years, while for the non-RA population, these numbers were 18% and 35%, respectively. Consistent with the literature, females were more commonly affected by RA with 61% of RA subjects being female compared with 49% female population in the non-RA group. The percentage of the population belonging to the lower income group (annual income <$25 K) was higher among RA subjects (42.6%) than non-RA subjects (30.8%).

**Table 1:** Comparison of demographic factors and comorbidities between study populations with RA and no arthritis. All *P* values are reported for *χ*^2^ tests performed on unweighted data.

Variables	Unweighted		Weighted		*P*
	*n* = 4,136		*N *= 163,540,241		
	No arthritis	RA	No arthritis	RA	
	*n *= 3,831 (%)	*n *= 305 (%)	*N *= 155,157,729 (%)	*N *= 8,380,512 (%)	
Age					<0.0001
≤40	1,781 (46.5)	30 (9.8)	78,833,815 (50.8)	1,148,070 (13.7)	
41–60	1,358 (35.5)	122 (40.0)	58,410,172 (37.6)	3,879,670 (46.3)	
> 60	692 (18.0)	153 (50.2)	17,915,741 (11.5)	3,352,772 (40.0)	
Gender					<0.0001
Male	1,957 (51.1)	120 (39.3)	79,796,903 (51.4)	3,371,900 (40.2)	
Female	1,874 (48.9)	185 (60.7)	75,362,826 (48.6)	5,008,613 (59.8)	
Income					<0.0001
< $25,000	1,180 (30.8)	130 (42.6)	35,684,140 (23.0)	3,126,096 (37.3)	
$25,000–75,000	1,471 (38.4)	109 (35.7)	60,118,646 (38.8)	3,187,188 (38.0)	
> $75,000	819 (21.4)	41 (13.4)	50,108,916 (32.3)	1,474,647 (17.6)	
NA	361 (9.4)	25 (8.3)	9,248,027 (6.0)	592,582 (7.1)	
Comorbidities					
Depression	246 (6.4)	61 (20.0)	8,299,320 (5.4)	1,576,573 (18.8)	<0.0001
Hypertriglyceridemia	493 (12.9)	63 (20.7)	18,444,010 (11.9)	1,610,680 (19.2)	<0.0001
Obesity	1,292 (33.7)	157 (51.5)	448,086,197 (31.0)	4,529,270 (54.1)	<0.0001
Stroke	64 (1.7)	29 (9.5)	1,679,696.2 (1.1)	691,128.8 (8.3)	<0.0001
Diabetes	319 (8.3)	84 (27.5)	8,787,123 (5.7)	2,003,661 (23.9)	<0.0001
Hypertension (sys.)	755 (20.7)	124 (40.7)	22,579,296 (14.6)	2,936,593 (35.0)	<0.0001
Hypertension (dias.)	593 (15.5)	42 (13.8)	21,935,832 (14.1)	1,438,295 (17.2)	<0.0001

**Figure 2:**
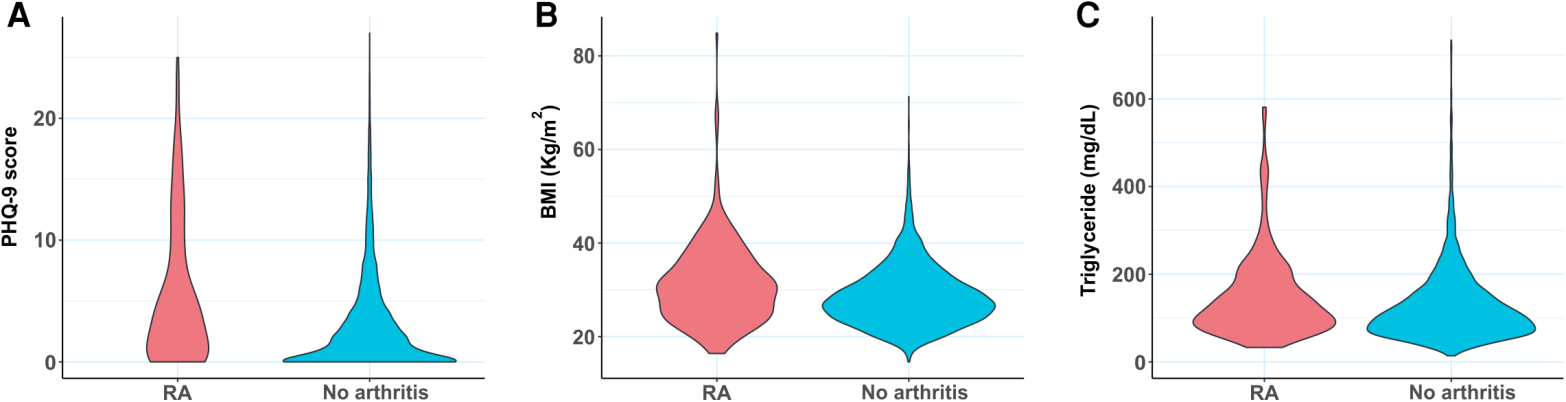
Violin plots comparing the distribution of (**A**) PHQ-9 score, (**B**) BMI, and (**C**) triglyceride level between RA subjects and control population with no arthritis. BMI, body mass index; PHQ-9, Patient Health Questionnaire (9 questions related to patient health).

Individuals with RA were found to have a stronger association with common comorbidities including hypertension, stroke, hypertriglyceridemia, depression, diabetes, and obesity (Table 1; *P* < 0.0001 for all). The distributions of the three RA comorbidity indicators considered here (PHQ-9 score, BMI, and triglyceride level) were further visualized among RA and non-RA populations using violin plots (Figure 2). These plots illustrate the probability density function and are often preferred to visualize the distribution of sample data over other commonly used plots such as box plots, which display the summary of the data only. For all three measurements, we observed a distinct difference in the plot shape between the two groups with a higher probability density at higher values for the RA population. This observation indicates that RA is associated with a distributional shift to higher values of PHQ-9 score, BMI, and triglyceride level.

**Figure 3:**
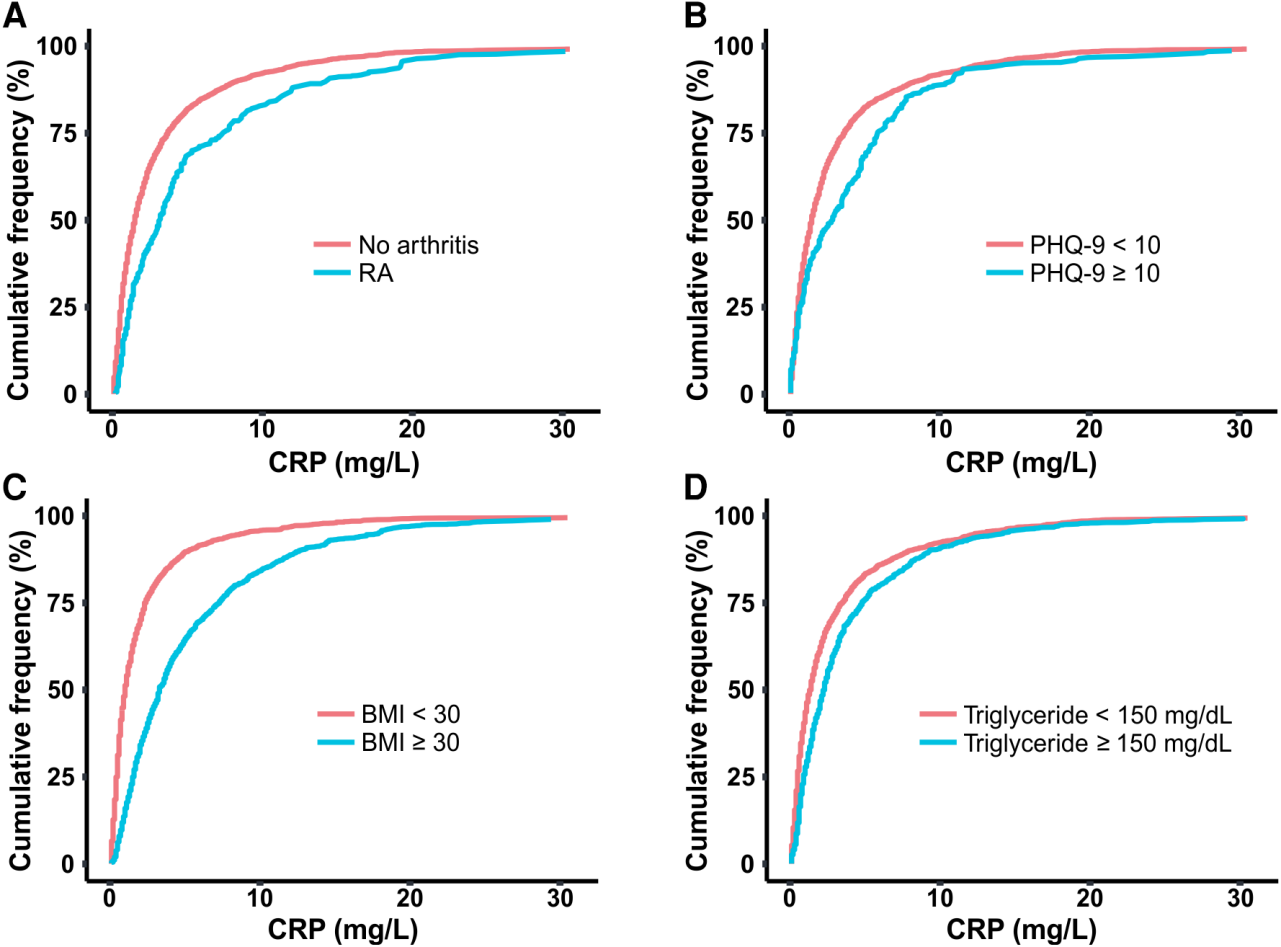
Cumulative distribution plots comparing CRP level among populations of (**A**) RA and no arthritis, (**B**) moderate-severe and no-mild depression (PHQ-9 score cut off at 10), (**C**) obesity and no obesity (BMI cut off at 30 kg/m^2^), (**D**) hypertriglyceridemia (HTG) and no HTG (triglyceride concentration cut off at 150 mg/dl). CRP, C-reactive protein; BMI, body mass index; PHQ-9, Patient Health Questionnaire (9 questions related to patient health).

As tissue inflammation is a hallmark of RA, we compared serum levels of the inflammatory marker CRP between RA and non-RA populations. The cumulative distribution plot of CRP showed a clear right shift for the RA population when compared with the plot for the control population, suggesting higher inflammation in general (Figure 3A). For example, according to this plot, 70.2% of the control population has CRP levels below 3.0 mg/L value, while only 47.7% of the RA population falls below this concentration. The reported association of chronic low-grade inflammation with depression, obesity, and HTG also motivated us to explore any connection of CRP with PHQ-9 score, BMI, and triglyceride level (37, 38). We divided each of these three variables into two categories based on medically relevant threshold values, separating subjects with depression (PHQ-9 score ≥10), obesity (BMI ≥30), and HTG (triglyceride ≥150 mg/dl) from subjects without these conditions (Figures 3B–D) (20, 33, 35). Cumulative distribution plots of CRP revealed higher values in populations with depression, obesity, and HTG with the difference being most prominent for obesity. The analysis of CRP level thus supports the reported connection of inflammation with RA as well as the comorbid conditions considered in this study.

**Figure 4:**
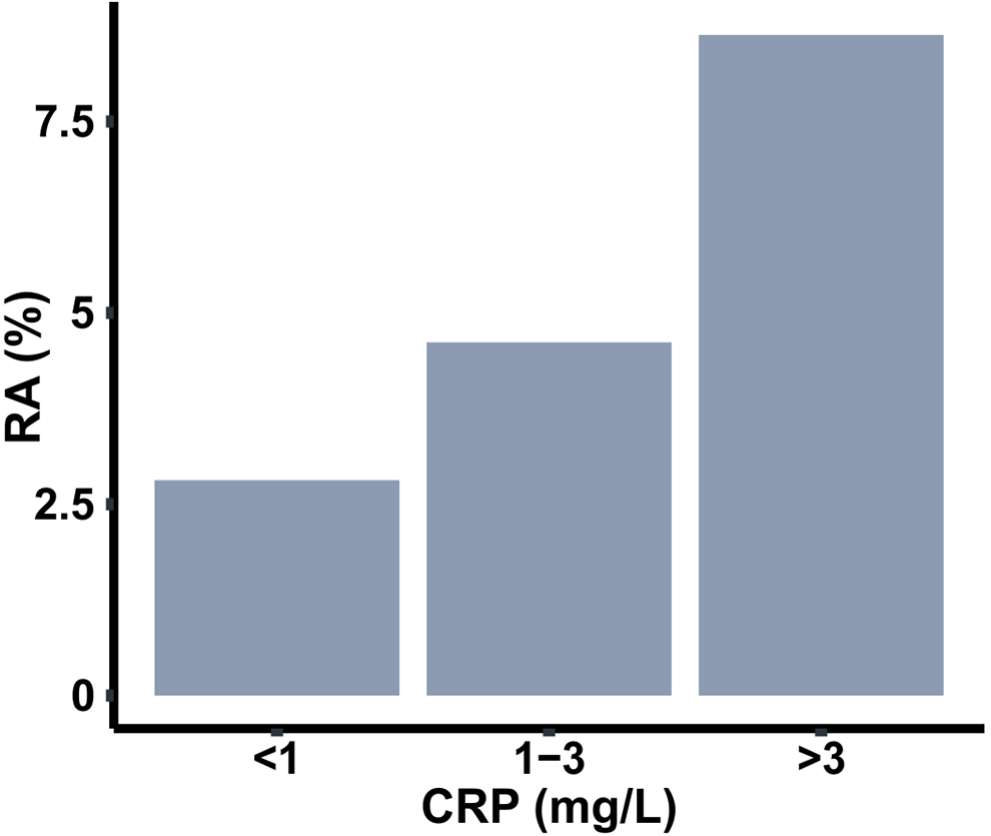
Prevalence of RA among populations with three different ranges of C-reactive protein (CRP).

Next, we explored how the coexistence of these comorbidities influences the association with RA. Furthermore, since elevated CRP values were observed with both RA as well as these comorbid conditions, we were interested to understand whether the association is impacted by the inflammatory state. To investigate this question, we divided the sample population into three categories based on CRP levels of low (<1 mg/L), medium (1–3 mg/L), and high (>3 mg/L) (36). This categorization divided the total sample population into three groups of comparable size. We observed the percentage of RA subjects progressively increase from low to high CRP group (Figure 4), further confirming the connection of inflammation to RA.

**Figure 5:**
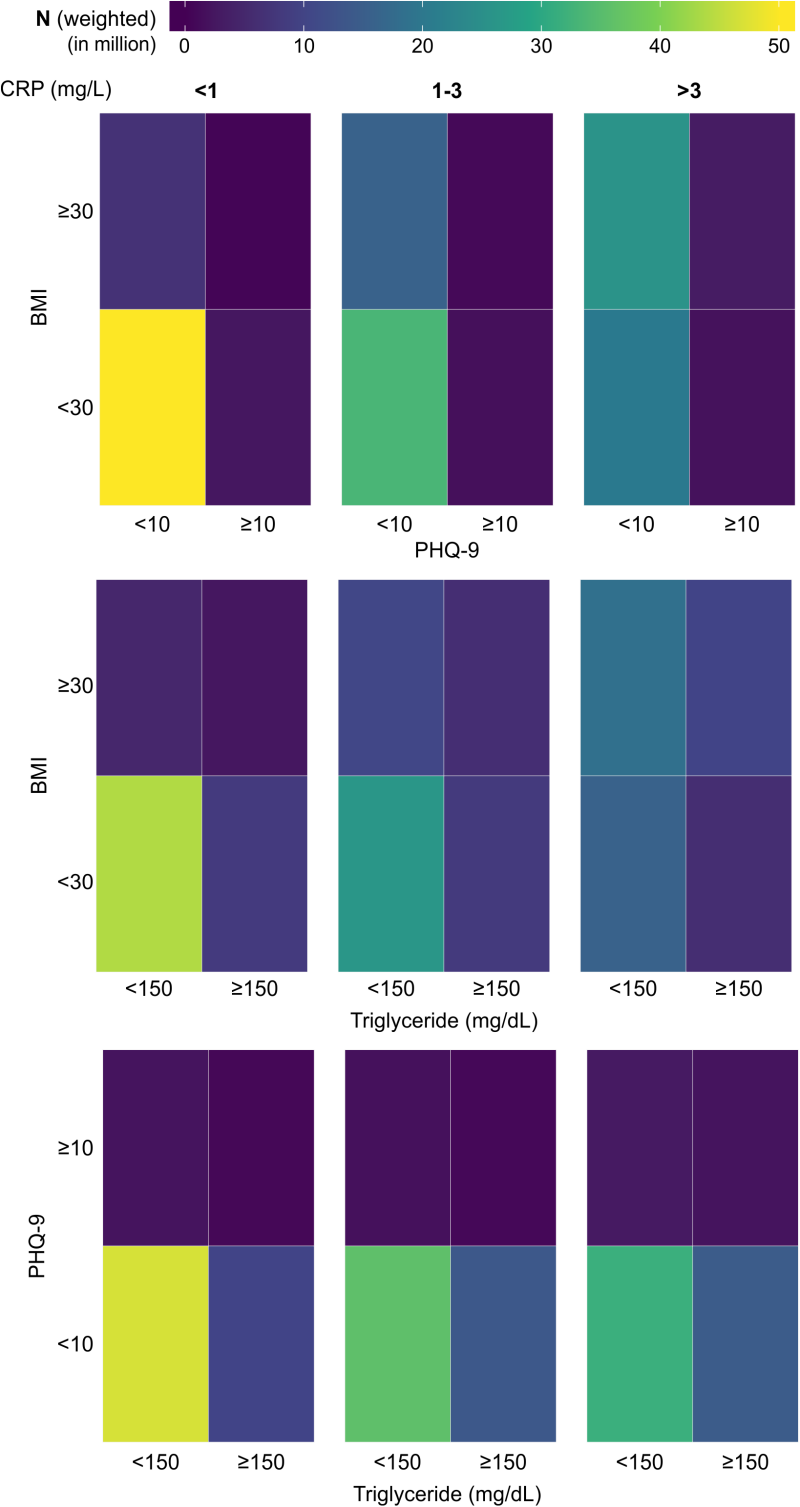
Tile plots showing the presence of depression, obesity, and hypertriglyceridemia (HTG) in the population, considering any two variables at a time. The population is further subdivided into three categories based on CRP level (<1 mg/L, 1-3 mg/L, and >3 mg/L). CRP, C-reactive protein; BMI, body mass index; PHQ-9, Patient Health Questionnaire (9 questions related to patient health).

Since the subjects with depression, obesity, and HTG showed higher CRP values in cumulative distribution plots, we first studied their prevalence across the CRP groups. Specifically, we were interested to know whether the presence of one of these conditions could influence the coexistence of another condition. To explore this, we created 2 × 2 tile plots (which display values in a grid of rectangular tiles) for each of the CRP ranges under consideration, taking any two comorbid conditions at once (Figure 5). For the ease of visualization of prevalence and identify any association between two conditions, the plots were color mapped where the color of a tile in the grid represents the magnitude of the number with purple being the lowest and yellow being the highest. Colormap representation of the sample population in these pair-wise tile plots revealed that the majority of the population in the low CRP group consisted of the absence from either condition. The proportion of subjects with obesity increased substantially at medium and high CRP levels; HTG showed a similar but weaker trend, but such a change was not conspicuous for depression. Next, we checked the pattern of coexistence between any two conditions, where a higher propensity for coexistence will be reflected by a relatively higher proportion of subjects in the upper right quadrant of a 2 × 2 tile plot. The tile plots did not indicate any preferential association across the CRP groups.

**Figure 6:**
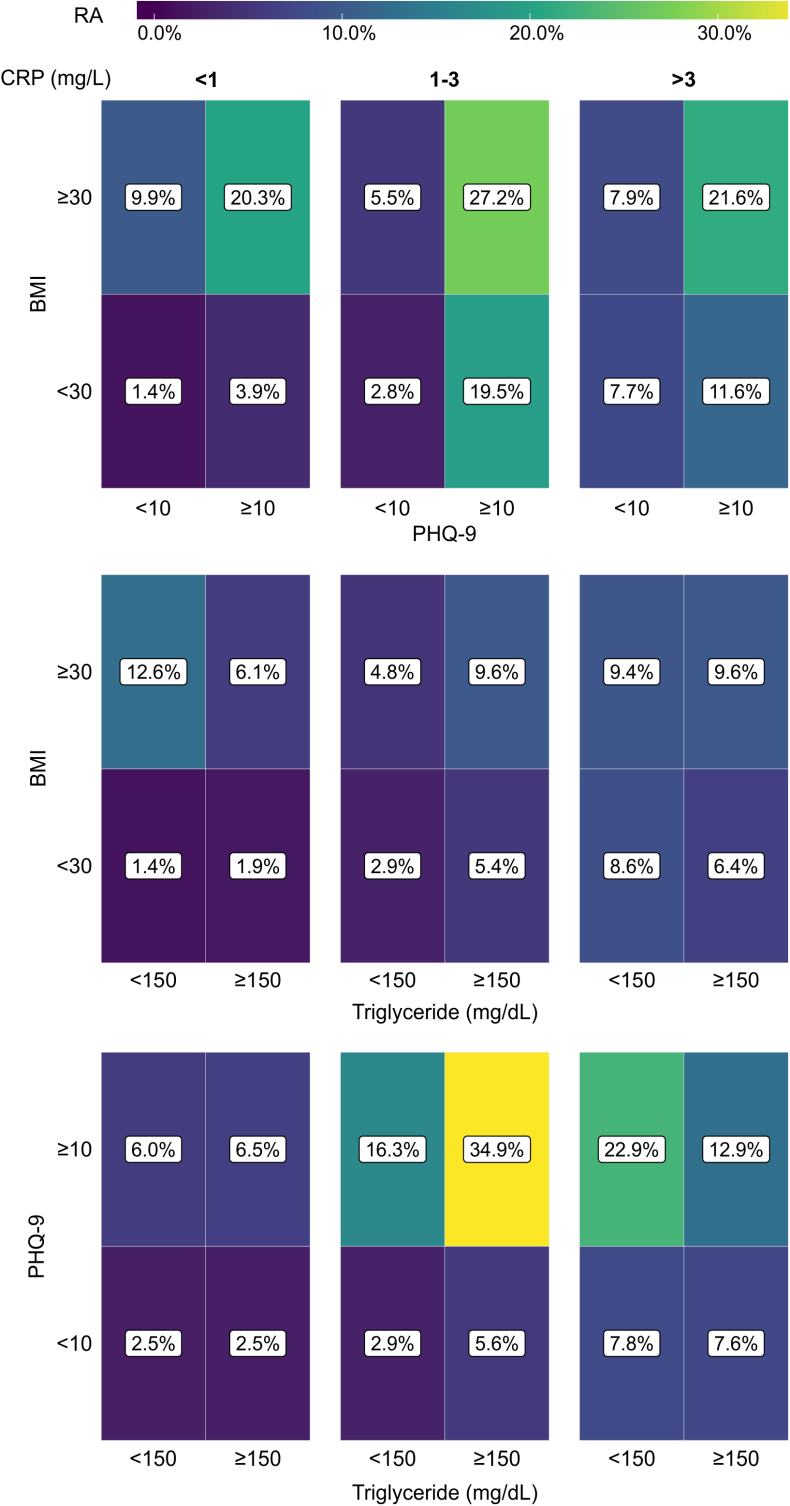
Tile plots showing the prevalence of RA (%) in the population with coexistent presence of depression, obesity, and hypertriglyceridemia (HTG), considering any two comorbidities at a time. The population is further subdivided into three categories based on CRP level (<1 mg/L, 1-3 mg/L, and >3 mg/L). CRP, C-reactive protein; BMI, body mass index; PHQ-9, Patient Health Questionnaire (9 questions related to patient health).

We took a similar approach to examine whether the coexistence of any two conditions among depression, obesity, and HTG could influence the association with RA. Similar to Figure 5, 2 × 2 tile plots were created where the percentages of RA subjects in subpopulations with or without any two comorbidities are shown (Figure 6). The colormap in the tile plots indicates the RA prevalence within a subpopulation with purple representing a low prevalence and yellow representing a high prevalence. The plots revealed distinct differences in RA prevalence depending upon the presence of comorbidities and CRP levels. Depression or obesity was found to be associated with a noticeable increase in RA prevalence, but such a relationship was not observed for HTG. Furthermore, for obesity, the effect was more prominent for the subjects in the low and medium CRP group, while for depression, a stronger effect was observed in the medium or high CRP group. Additionally, we observed a substantial increase in RA prevalence among subjects who have depression along with obesity or HTG. This effect was observed across all CRP ranges for obesity with a slightly stronger effect at the medium level of CRP (1–3 mg/L); for HTG, the effect was prominent only at the medium CRP level. For example, when depression and obesity were considered at the medium CRP range, the prevalence of RA in subjects with depression and obesity were 19.5% and 5.5%, respectively; however, RA prevalence increased to 27.2% for the population with both depression and obesity. Similarly, a high RA prevalence of 34.9% was observed among subjects with depression and HTG at the medium CRP level, while the prevalence of RA among subjects with depression and HTG alone were 16.3% and 5.6%, respectively. Interestingly, at a high CRP level (>3 mg/L), the coexistence of HTG among the subjects with depression had a lower prevalence of RA (12.9%) than subjects with depression but not HTG (22.9%).

To make a quantitative interpretation of the findings in Figure 6, we conducted three-way *χ*^2^ tests of independence (Table 2). We find the proportion of subjects with RA to be significantly different (*P* < 0.05) for all situations, except when depression and HTG were considered at the low level of CRP. The standardized residuals were then used to understand the contribution of each categorical combination to the *χ*^2^ test result. Corroborating the observation from Figure 6, high standardized residual values were found for RA positivity when depression coexisted with obesity at all CRP levels or with HTG in the medium level of CRP. For example, among the subjects with depression and HTG in the medium level of CRP, the standardized residuals for RA positive and negative were 6.35 and −0.76, respectively; in contrast, the standardized residual values for RA positive and negative were 0.97 and −0.34 when depression with the absence of HTG was considered in the same CRP range. Furthermore, when the presence of the same pair of conditions was considered at the high CRP level, the standardized residual values for RA positive and negative were 1.75 and 1.28, respectively. Thus, the *χ*^2^ test results confirm the synergistic relationship of depression with obesity and HTG for the association with high RA prevalence, and the potential contribution of the inflammatory states of the subjects to underlie such association.

**Table 2:** Standardized residuals from three-way *χ*2 tests to study the association between RA and the comorbidities depression, obesity, and hypertriglyceridemia, measured at three different plasma CRP levels. Weighted data, scaled down to the sample size of the unweighted data, were used for the analysis.

BMI	PHQ-9	RA	Standardized Residuals		
			Low CRP (<1 mg/L)	Medium CRP (1–3 mg/L)	High CRP (>3 mg/L)
			*P* < 0.001	*P *< 0.001	*P *< 0.001
≥30	≥10	Yes	4.27	4.63	4.59
		No	−0.29	−0.56	−0.09
	<10	Yes	5.27	1.04	−0.90
		No	−0.95	−0.32	−0.15
<30	≥10	Yes	0.57	4.06	0.05
		No	−0.24	−1.19	−1.54
	<10	Yes	−2.42	−2.28	−0.68
		No	0.43	0.56	0.70
Triglyceride (mg/dl)	BMI	RA	*P *< 0.001	*P* < 0.001	*P *< 0.001
≥150	≥30	Yes	2.79	3.90	0.95
		No	4.04	2.58	1.36
	<30	Yes	−0.90	0.08	−1.60
		No	−1.52	−2.40	−1.39
<150	≥30	Yes	5.53	−0.09	0.17
		No	−2.99	−2.11	−1.19
	<30	Yes	−2.11	−1.67	0.16
		No	1.12	1.84	1.25
Triglyceride (mg/dl)	PHQ-9	RA	*P *= 0.156	*P *< 0.001	*P *= 0.037
≥150	≥10	Yes	1.37	6.35	1.75
		No	1.14	−0.76	1.28
	<10	Yes	−0.28	0.97	−0.88
		No	−0.26	−0.34	−0.31
<150	≥10	Yes	1.54	3.13	3.39
		No	−0.89	−1.06	−2.31
	<10	Yes	−0.35	−2.12	−0.88
		No	0.20	0.54	0.68

## 4. Discussion

In this study, we compared the association of RA with three common chronic conditions depression, obesity, and HTG, with a specific focus on any changes in their association due to the coexistence of another condition. We observed that the prevalence of RA among subjects with depression was strongly increased in the presence of obesity or HTG. Our analysis also showed that the degree of synergistic association is strongly dependent on the inflammatory state of the body with the effect being maximum at the medium level of CRP (1–3 mg/L).

The relationship between depression and RA has been extensively studied since it is one major comorbid condition for RA and significantly impacts the disease outcome, including mortality (12–14). Recent studies suggest a bidirectional association between these two disease conditions, implying that subjects suffering from depression are also more likely to develop RA (15, 39). Our study indicates that the proportion of RA among subjects with depression is considerably higher when the subjects also had obesity or HTG. Interestingly, even though RA prevalence was higher among subjects with obesity, the coexistence of HTG did not further increase the prevalence. Thus, the observed synergistic association is specific to depression among the conditions studied here. Due to the cross-sectional nature of the data, we can't conclude whether the presence of obesity or HTG poses an increased risk of developing RA among the subjects with depression. Also, the size of our study population did not allow us to study if the RA prevalence is further impacted when both obesity and HTG are present in subjects with depression as the number of subjects with all three conditions was too low to perform any meaningful analysis. It is to be noted that the prevalence of both RA and depression substantially varies with demographic factors such as age, sex, or ethnicity; thus, these variables could play a confounding role in the analysis, and the extent of synergistic effect observed with obesity and HTG could vary considerably when specific subpopulations are considered. Regardless, our findings imply the need for additional attention when evaluating depression with obesity or HTG and warrant a longitudinal study to evaluate the risk of RA in these subjects.

One key finding from our analysis is that the observed association is dependent on CRP level, a marker of inflammation. CRP is widely used in clinical settings to evaluate RA disease activity (40), and we have also found RA prevalence to increase with the CRP level. The reported connection of inflammation with depression, obesity, and HTG is corroborated in our analysis where CRP levels were found to be higher in these conditions compared with the control populations (27, 28, 41, 42). Interestingly, the CRP levels at which obesity and HTG influenced the association of depression with RA were found to be distinct. While the influence of BMI was observed across all CRP levels (low, medium, and high), the effect of HTG was prominent only at the medium CRP level. Furthermore, HTG was found to decrease the prevalence of RA among subjects with obesity at low CRP level and subjects with depression at high CRP level. Overall, these results suggest a potentially important role of immunity in determining the interactions between depression, obesity, and HTG on their association with RA; however, the nature of such interactions is not well understood from the current results based on CRP. Measurement of CRP can be useful to quickly assess the systemic inflammatory state, but it is highly nonspecific, and its serum level can be elevated by a broad array of conditions that induce inflammation, including infections and tissue damage. Inflammation involves a complex interplay of large numbers of inflammatory mediators and specific pathways are known to be predominant in the pathogenesis and manifestation of certain disease conditions. Therefore, a deeper exploration of the inflammatory pathways is necessary to better understand the associations observed here.

In recent years, there is an emerging interest in the prediction of RA from a set of known risk factors. Such interest is primarily motivated by the detection of the disease at the “pre-RA” period when the subject is yet to clinically develop RA symptoms, but the presence of RA-related autoantibodies can be detected in the blood (43, 44). Diagnosis in the pre-RA period, although challenging, can be extremely advantageous as intervention started at an earlier stage can considerably slow down the disease progress and bring favorable outcomes (43). Models constructed toward RA prediction considered a wide range of potential risk factors under consideration, including genetic, socio-demographic, and environmental factors, comorbidities, and results of clinical and laboratory examination (45–49). However, interactions between these factors were not included in most of these models—except when a specific interaction is already known or suspected (45, 48)—as that would otherwise exponentially increase the computational cost (49). In this context, our observation of synergistic interactions of depression with obesity and HTG could provide important insights to incorporate these specific interactions in a prediction model to improve prediction accuracy.

Although our analysis provides important insights into how the coexistence of certain chronic conditions can influence their association with RA, there are a few limitations in this study. First, the cross-sectional nature of the data only allows the inference on the association between variables rather than concluding on their causal relationship. Given the subjects of depression are at higher risk of RA, our findings highlight the need for a longitudinal study investigating the impact of the coexistence of depression with BMI or obesity on RA incidence. Second, the self-reported diagnosis of RA used in this study is prone to overestimate RA prevalence due to the increased chance of false-positive diagnosis, mostly coming from erroneous inclusion of other forms of arthritis. While well-established diagnostic criteria of RA such as one provided by the American College of Rheumatology (ACR) and European League Against Rheumatism (EULAR) (50) should be used for clinical decision-making, self-reported RA is considered to have an acceptable level of accuracy for epidemiological studies (51). Since NHANES provides a large nationwide dataset with a very well-structured sampling design, we believe that even with these limitations, it provides suitable data to conduct preliminary analysis. Another potential limitation of the current study arises from the dichotomization of variables such as BMI, PHQ-9 score, and triglyceride concentration. While the conversion into categorical variables facilitated studying the association of RA with the presence/absence of comorbid conditions and provided sufficient sample size for statistical analysis, such transformation is associated with loss of information, reduced statistical power, lower sensitivity, and an increased risk of biased conclusion from the choice of cut off value. Future studies should be conducted on a larger sample population where the association between variables could be investigated without dichotomization. Finally, CRP is a good marker for the initial screening of the inflammatory state but has a limited value in probing the potential crosstalk between inflammatory signaling pathways. Therefore, to understand the molecular underpinnings of the synergistic interactions observed here, measurement of a broader panel of inflammatory markers (e.g., TNF-α, IL-1, IL-6) should be incorporated in future studies.

## Data Availability

Publicly available datasets were analyzed in this study. This data can be found here: https://wwwn.cdc.gov/nchs/nhanes/.
